# Supplementary material for: NETosis and thrombosis in vaccine-induced immune thrombotic thrombocytopenia
Source: Nat Commun. 2022 Sep 5;13:5206. doi: 10.1038/s41467-022-32946-1 (PMC9441824; doi:10.1038/s41467-022-32946-1)
Supplement: Supplementary file 1 — Supplementary Information [file 41467_2022_32946_MOESM1_ESM.pdf]

NETosis and thrombosis in vaccine-induced immune thrombotic thrombocytopenia

Halina HL Leung[1§], Jose Perdomo[1§], Zohra Ahmadi[1], Shiying S Zheng[1,2], Fairooj N Rashid[3,4], Anoop Enjeti[5,6], Stephen B Ting[7], James JH Chong[3,4,8], Beng H Chong[1,2*]

Supplementary Information

Neutrophil and monocyte gating and monocyte ETs

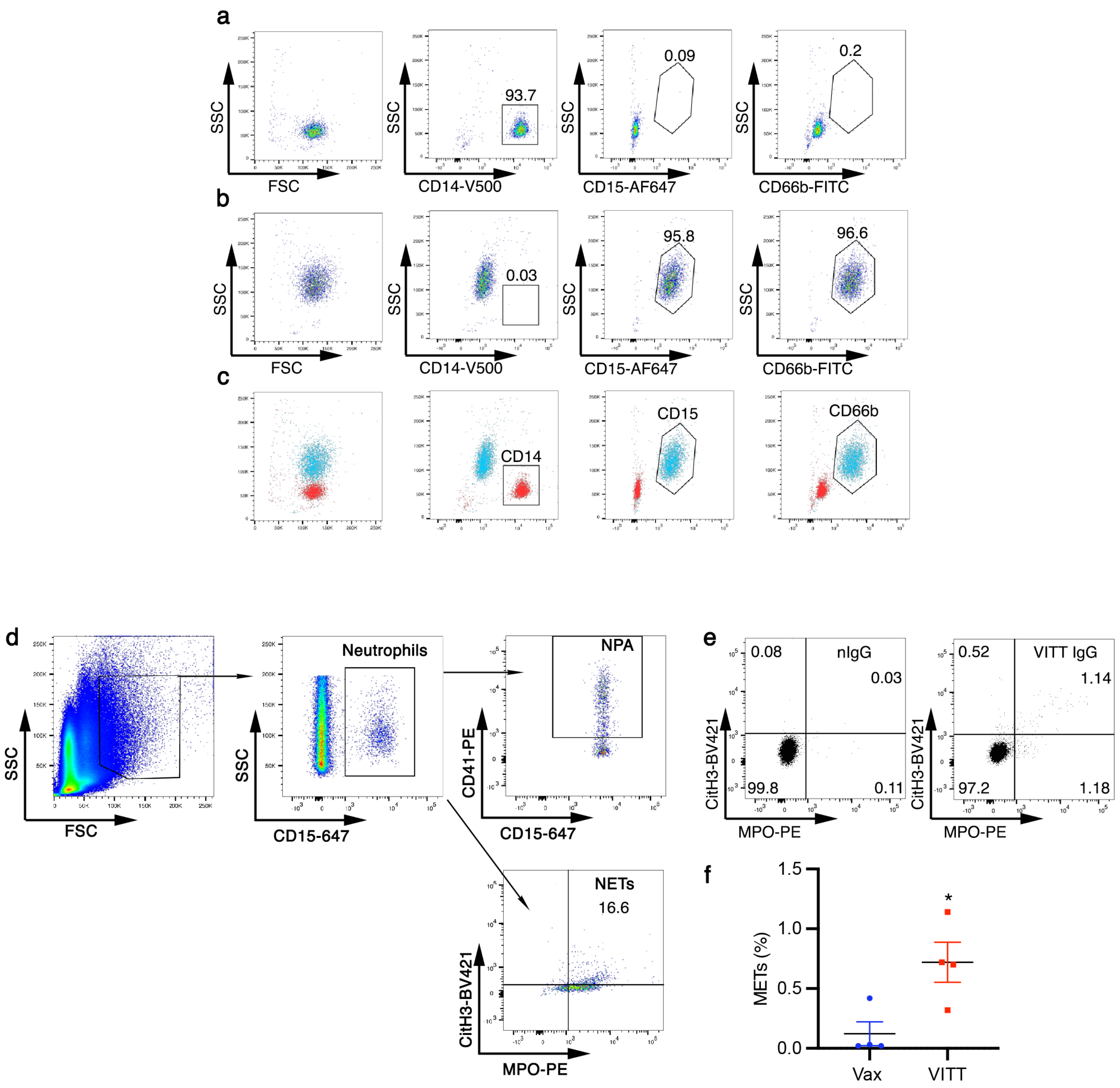

Supplementary Figure 1

Supplementary Figure 1. Representative flow cytometry density plots using fresh blood to show the neutrophil and monocyte populations. **a** $CD14^+CD15^-CD66b^-$ was defined as monocytes. **b** $CD14^-CD15^+CD66b^+$ population was defined as neutrophils. The numbers over the gates indicate percentages of the total population. **c** Backgating shows no overlap of these populations. Monocytes, red; neutrophils, blue. **d** Gating strategy. Left panel shows side scatter versus forward scatter of whole blood and the gated population. Neutrophils ($CD15^+$ cells) were gated to analyse NPA ($CD41^+CD15^+$ aggregates) and NETs ($CitH3^+MPO^+$ cells). This example shows staining of fresh blood from VITT patient. **e** Representative flow cytometry density plots of purified monocytes treated with VITT IgG and PF4. Monocyte extracellular traps defined as $CitH3^+MPO^+$ cells. Percentage of gated events in $CD14^+$ population is indicated in the upper right quadrant. **f** Quantification of monocyte ETs following treatment of isolated monocytes purified from healthy donor blood with VITT IgG and PF4 (n = 4; *p = 0.022). Statistics: Unpaired two-tailed t test. Source data are provided in the Source Data file.

IgG analysis and affinity purified anti PF4

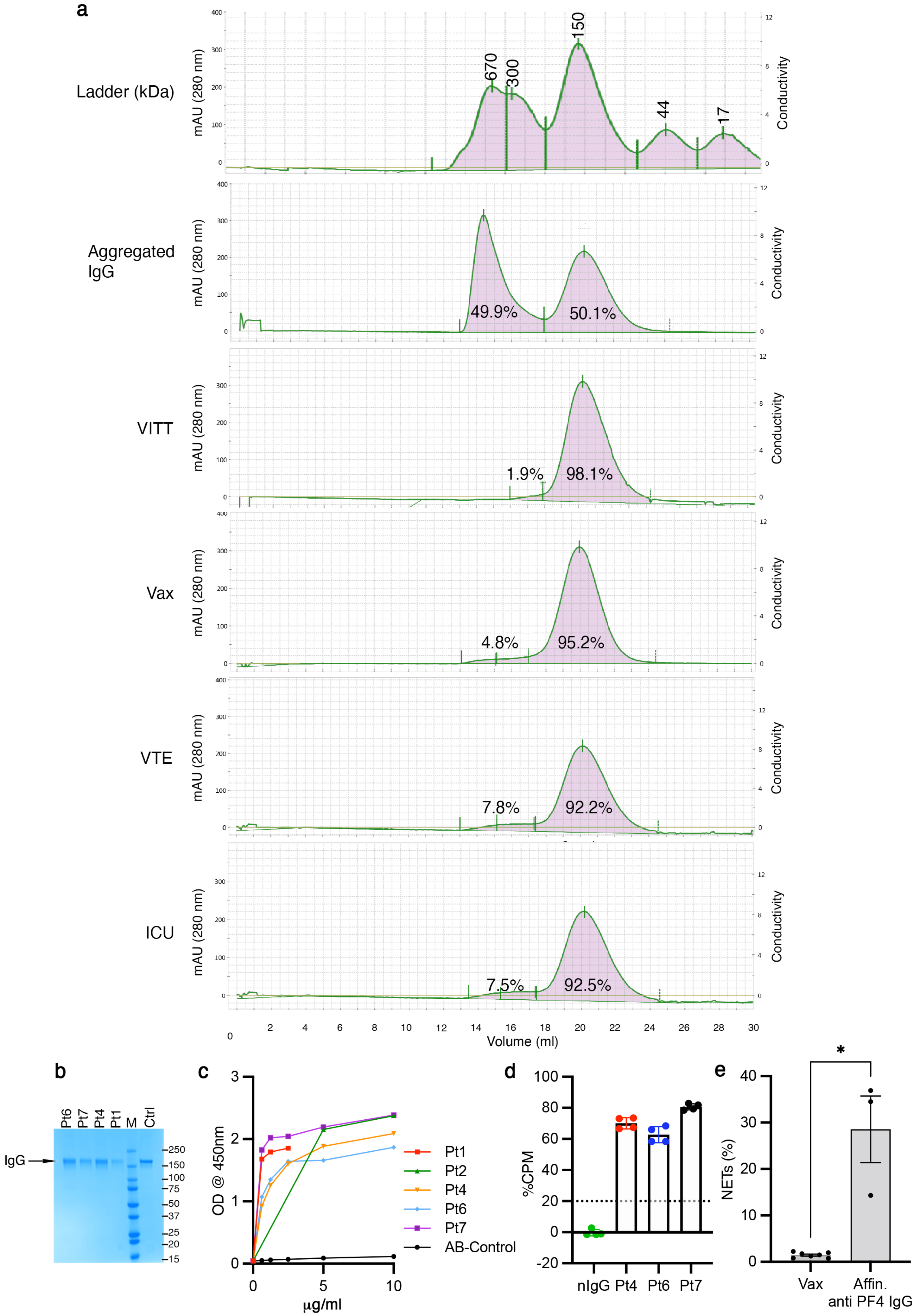

Supplementary Figure 2

Supplementary Figure 2. a Size exclusion chromatography traces for purified IgG. Ladder shows molecular weight in kDa (top). Aggregated IgG was used to show large aggregates (over 600 kDa peak) and monomeric IgG (150 kDa peak). The traces show that IgG from VITT, vaccine control, VTE and ICU donors was monomeric, with small percentages of aggregates. **b** Non-reducing SDS gel showing affinity purified anti PF4 IgG from VITT patients (arrow). Control line (Ctrl) shows purified total IgG as comparison. Experiment was repeated independently 3 times. **c** PF4 ELISA experiment using different concentrations of affinity purified anti PF4 IgG from VITT patients. IgG purified from pooled AB group serum was used as control (n = 2). **d** ^{14}C-serotonin release assay for affinity purified anti PF4 IgG in the presence of PF4 (10 µg/mL). Each dot represents the mean of assays done in triplicate. The cut-off was set at 20% CPM (n = 4). **e** Purified neutrophils treated with Vax IgG or affinity purified anti PF4 IgG from VITT patients in the presence of PF4 (10 µg/mL). NETs induction was identified as $CitH3^{+}MPO^{+}$ in $CD15^{+}$ population (Vax n = 7; Affin. anti PF4 IgG n = 3; *p = 0.016). Data presented as (**c**-**d**) mean ± SD; (**e**) mean ± SEM. Statistics: Mann-Whitney test, two-tailed. nIgG, normal IgG; Vax, vaccine control; VTE, venous thromboembolism; ICU, intensive care unit; CPM, counts per minute; Pt, patient; M, molecular weight marker. Source data are provided in the Source Data file.

Thrombosis in vitro and in vivo

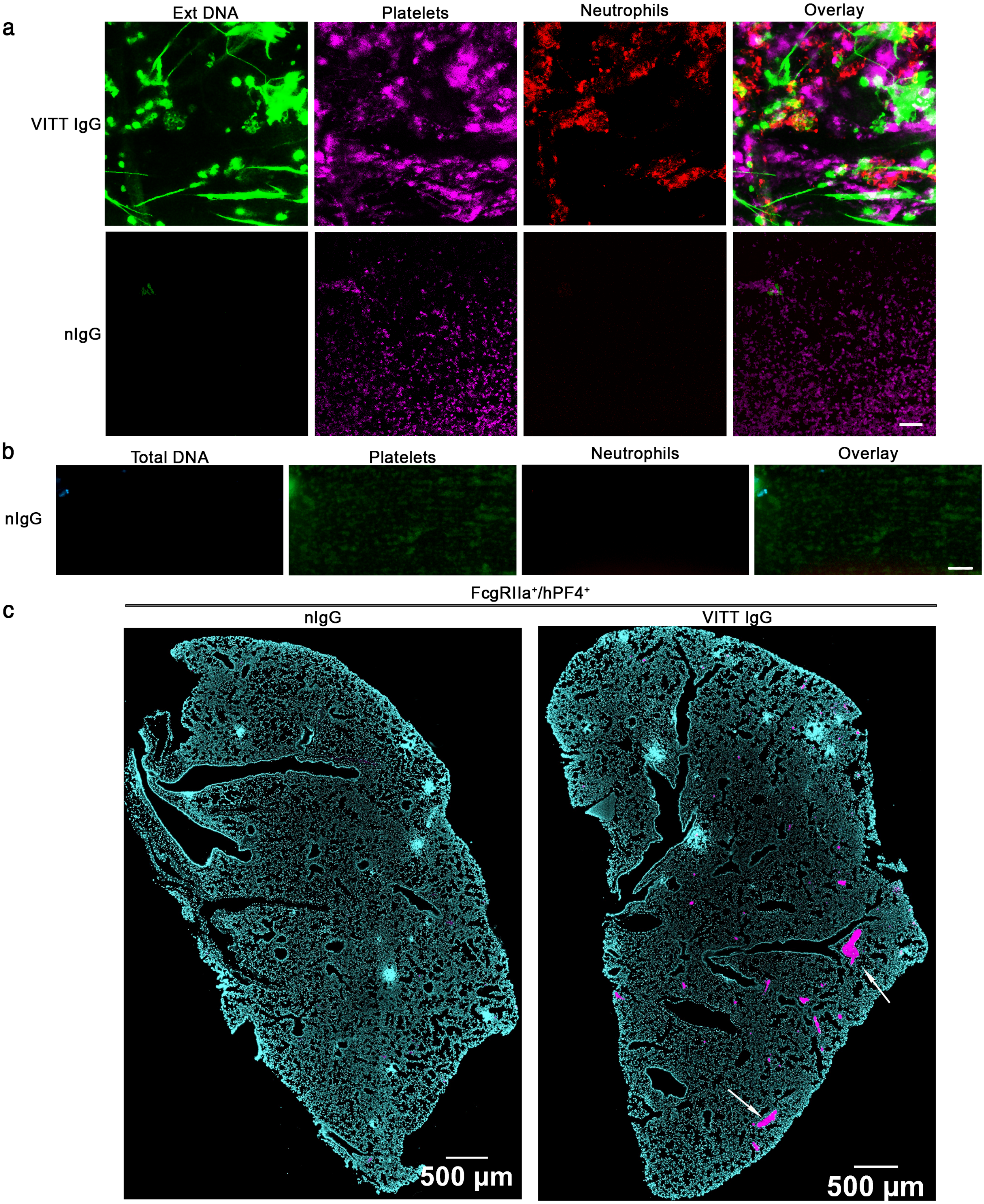

Supplementary Figure 3

Supplementary Figure 3. a VITT IgG and thrombosis. Healthy donors' blood treated with VITT IgG was flowed in vWf-coated microchannels. Extracellular DNA was stained with Sytox green (green), platelets with anti-CD41 AF647 (magenta) and neutrophils with anti-CD15 AF594 (red). Thrombi were imaged with a confocal laser scanning microscope (Leica TCS SP8 running Leica's LAS X software) with a 63x oil immersion objective. Scale bar 20 µm. **b** Healthy donors' blood treated with normal IgG was flowed in vWf-coated microchannels. Total DNA was stained with Hoechst 33342 (blue), platelets with anti-CD41-FITC (green) and neutrophils with anti-CD15 AF594 (red). Scale bar 50 µm. **c** Fluorescent images of lung lobes from mice treated with VITT IgG or control IgG. DAPI-stained nuclei (blue), platelet-rich thrombi (magenta, white arrows). Scale bar 500 µm. Experiment was repeated independently (**a**-**b**) at least 5 times; (**c**) 3 times.

Number of clots and systemic response

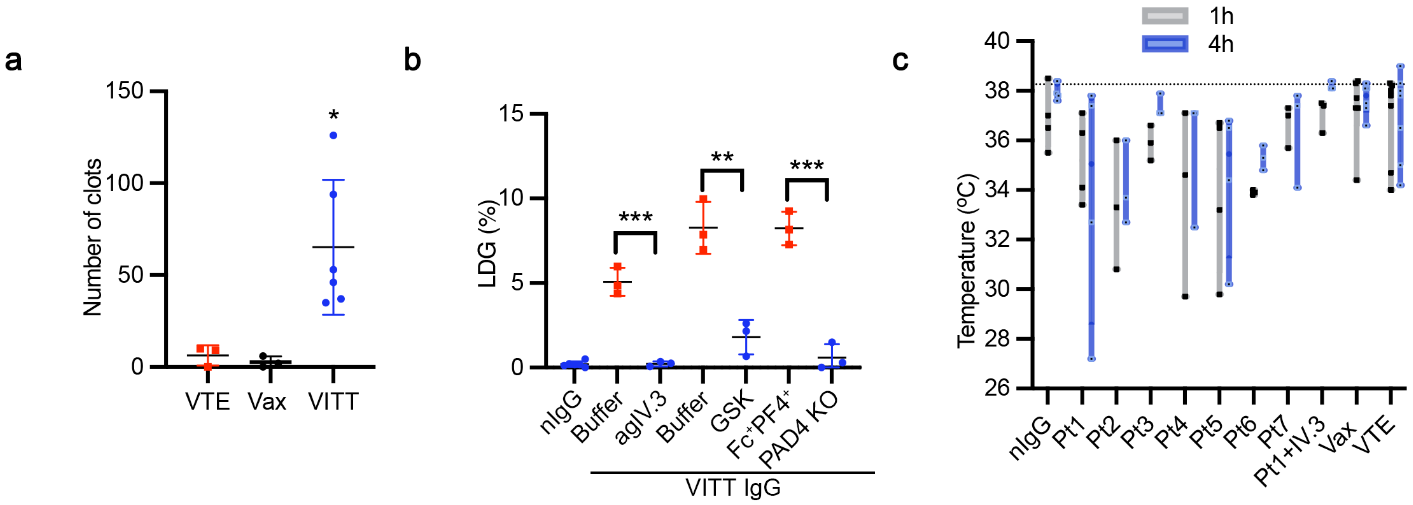

Supplementary Figure 4

Supplementary Figure 4. **a** Enumeration of the number of clots in lung sections from mice treated with purified IgG from venous thromboembolism (VTE, *n* = 3), vaccine control (Vax, *n* = 3) and VITT patients (*n* = 6). Each dot represents one mouse. *p = 0.017 **b** Level of low density granulocytes (LDG) in blood from mice following the treatments indicated in the Figure (*n* = 3, except nIgG *n* = 6; VITT vs VITT+agIV.3 *** p = 0.006; VITT vs VITT+GSK ** p = 0.0037; VITT vs PAD4 KO *** p = 0.0005). Data presented as mean ± SD (**a**-**b**). Statistics: (**a**) Kruskal-Wallis with Dunn's correction; (**b**) unpaired two-tailed t test. nIgG, normal IgG; PAD4 KO, PAD4 knockout FcγRIIa+/hPF4+ mice. **c** Changes in temperature following the treatments indicated in the figure. Dotted line represents the mean temperature of mice before treatment (38.3°C, *n* = 30). Source data are provided in the Source Data file.

hPF4 concentration in transgenic mice and proposed mechanism of thrombosis in VITT

a

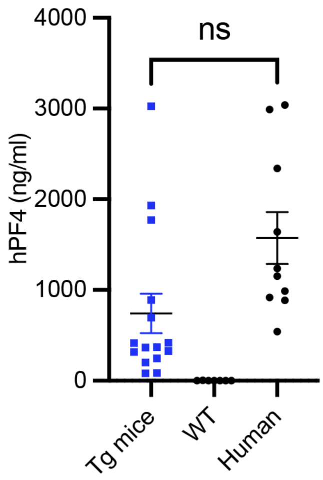

b

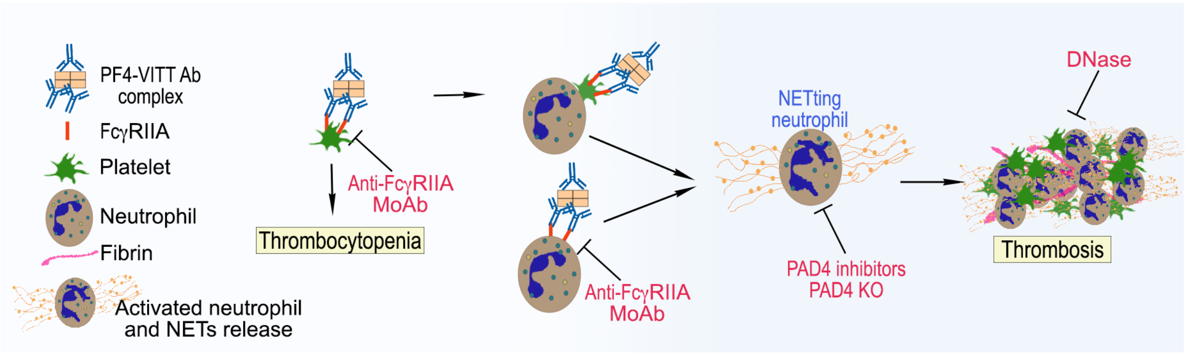

Supplementary Figure 5

Supplementary Figure 5. a Human PF4 levels in FcγRIIa/hPF4 mice (*n* = 15), wild type C57/Bl6 mice (*n* = 7) and humans (*n* = 10) were measured by ELISA. Data presented as mean values ± SEM. Statistics: Kruskal-Wallis test. **b** Model of mechanism of thrombosis and thrombocytopenia in VITT. Anti-PF4 antibodies from VITT patients form a complex with PF4 and interact with FcγRIIa. Interaction of the complex with platelets results in thrombocytopenia, which can be blocked with the monoclonal antibody IV.3. In the case of neutrophils, the interaction of the complex with FcγRIIa leads to NETs formation and subsequent thrombosis. Thrombosis can be blocked by neutralisation of FcγRIIa with IV.3 or by inhibition of NETosis using NETs inhibitor or in PAD4 knockout mice. In vitro, addition of DNase I disrupts thrombus formation. Tg, FcγRIIa$^+$/hPF4$^+$ mice; WT, wild type mice. Source data are provided in the Source Data file.

Supplementary Table 1: Clinical information of VITT patients. SRA, serotonin release assay; ELISA, enzyme-linked immunosorbent assay for anti-PF4 antibody; Fib, fibrinogen in g/L; Plt, platelet count x 10^9/L; D-dimer in mg/L; PE, pulmonary embolism; DVT, deep vein thrombosis.

VITT	Site of thrombosis	Lab test	Treatment
**1**	Portal vein, splenic vein, superior mesenteric vein thrombosis	Plt 70, D-dimer 114, Fib 3.0, ELISA positive, SRA positive	Bivalirudin, fondaparinux, warfarin, IVIg, methylprednisolone
**2**	Left central venous sinus thrombosis, intracranial haemorrhage	Plt 18, D-dimer >20, Fib 0.6, ELISA positive, SRA positive	Apixaban, argatroban, dabigatran, IVIg
**3**	Bilateral PE, DVT	Plt 21, D-dimer >20, Fib 2.1, ELISA positive, SRA positive	Fondaparinux
**4**	Portal vein, superior mesenteric vein thrombosis	Plt 93, D-dimer 56, Fib 1.9, ELISA positive, SRA positive	Apixaban, bivalirudin, fondaparinux, IVIg
**5**	PE, DVT	Plt 99, D-dimer >20, Fib 2.7, ELISA positive, SRA positive	Apixaban, fondaparinux
**6**	PE, DVT	Plt 8, D-dimer >10, Fib 1.7, ELISA positive, SRA positive	Bivalirudin, dexamethasone, IVIg
**7**	Central venous sinus thrombosis	Plt 138, D-dimer >10, Fib 2.9, ELISA positive, SRA positive	Bivalirudin, dabigatran, IVIg

Supplementary Table 2: Clinical information of controls. Control groups include patients with common venous thromboembolism (VTE) and critically ill patients admitted to intensive care units (ICU).

Group	No.	Diagnosis	Treatment
**VTE**	1	Left leg DVT	Enoxaparin, rivaroxaban
**VTE**	2	Left leg DVT	Enoxaparin, rivaroxaban
**VTE**	3	Antiphospholipid antibody syndrome, DVT, PE	Enoxaparin, warfarin
**VTE**	4	Right leg DVT	Enoxaparin, apixaban
**VTE**	5	Acute PE	Enoxaparin
**VTE**	6	PE, dermatomyositis	Enoxaparin, warfarin
**VTE**	7	Right leg DVT	Enoxaparin, rivaroxaban
**ICU**	1	Septicaemia, multiple myeloma	Hypotension - inotrope
**ICU**	2	T cell lymphoma, HLH, neutropenic septicaemia, cardiac arrest	Chemo, antibiotics, intubation
**ICU**	3	Polycystic kidney disease, renal transplant, polycystic liver, liver failure, CMV viraemia, sepsis	Antibiotics, dialysis, paracentesis
**ICU**	4	Severe COVID infection	Baricitinib
**ICU**	5	Subarachnoid haemorrhage, DVT	Neurological observation, then enoxaparin
**ICU**	6	Septicaemia, multiple myeloma	Antibiotics
**ICU**	7	COVID infection, Urosepsis/septicaemia, shock	Inotrope, antibiotics, sotrovimab
