# Supplementary material for: NETosis and thrombosis in vaccine-induced immune thrombotic thrombocytopenia
Source: Nat Commun. 2022 Sep 5;13:5206. doi: 10.1038/s41467-022-32946-1 (PMC9441824; doi:10.1038/s41467-022-32946-1)
Supplement: Supplementary file 2 — Reporting Summary [file 41467_2022_32946_MOESM2_ESM.pdf]

nature portfolio

Corresponding author(s): Beng Chong

Last updated by author(s): Jul 25, 2022

Reporting Summary

Nature Portfolio wishes to improve the reproducibility of the work that we publish. This form provides structure for consistency and transparency in reporting. For further information on Nature Portfolio policies, see our Editorial Policies and the Editorial Policy Checklist.

Statistics

For all statistical analyses, confirm that the following items are present in the figure legend, table legend, main text, or Methods section.

n/a	Confirmed	
☐	☒	The exact sample size (n) for each experimental group/condition, given as a discrete number and unit of measurement
☐	☒	A statement on whether measurements were taken from distinct samples or whether the same sample was measured repeatedly
☐	☒	The statistical test(s) used AND whether they are one- or two-sided<br>*Only common tests should be described solely by name; describe more complex techniques in the Methods section.*
☐	☒	A description of all covariates tested
☐	☒	A description of any assumptions or corrections, such as tests of normality and adjustment for multiple comparisons
☐	☒	A full description of the statistical parameters including central tendency (e.g. means) or other basic estimates (e.g. regression coefficient) AND variation (e.g. standard deviation) or associated estimates of uncertainty (e.g. confidence intervals)
☐	☒	For null hypothesis testing, the test statistic (e.g. F, t, r) with confidence intervals, effect sizes, degrees of freedom and P value noted<br>*Give P values as exact values whenever suitable.*
☒	☐	For Bayesian analysis, information on the choice of priors and Markov chain Monte Carlo settings
☒	☐	For hierarchical and complex designs, identification of the appropriate level for tests and full reporting of outcomes
☒	☐	Estimates of effect sizes (e.g. Cohen's d, Pearson's r), indicating how they were calculated

Our web collection on statistics for biologists contains articles on many of the points above.

Software and code

Policy information about availability of computer code

Data collection	FACS DIVA v9, Living Image 4.5.5, Q-Imaging by Venaflux software, Leica Application Suite X v3.5.6.21594
Data analysis	FlowJo v10.8.1, ImageJ v2.1.0/1.53c, GraphPad Prism v8, Image-Pro Premier 9.1

For manuscripts utilizing custom algorithms or software that are central to the research but not yet described in published literature, software must be made available to editors and reviewers. We strongly encourage code deposition in a community repository (e.g. GitHub). See the Nature Portfolio guidelines for submitting code & software for further information.

Data

Policy information about availability of data

All manuscripts must include a data availability statement. This statement should provide the following information, where applicable:

- Accession codes, unique identifiers, or web links for publicly available datasets
- A description of any restrictions on data availability
- For clinical datasets or third party data, please ensure that the statement adheres to our policy

All data generated or analysed during this study are included in this published article (and its supplementary information files). A data availability statement is included in the manuscript.

Field-specific reporting

Please select the one below that is the best fit for your research. If you are not sure, read the appropriate sections before making your selection.

☒ Life sciences ☐ Behavioural & social sciences ☐ Ecological, evolutionary & environmental sciences

For a reference copy of the document with all sections, see nature.com/documents/nr-reporting-summary-flat.pdf

Life sciences study design

All studies must disclose on these points even when the disclosure is negative.

Sample size	Sample size was estimated based on previous publications demonstrating statistical significance in measuring similar outcomes. Post hoc analysis (G*Power, Version 3.1.9.3) indicates a power of 99% at alpha 0.05 for the number of patients and controls used.
Data exclusions	No data were excluded.
Replication	Each individual healthy donor for in vitro experiments and each mouse used for animal experiments was considered a biological replicate. All samples were measured once as biologically independent samples. For all other experiments, assays were repeated on different days for independent replicates. All experimental findings were reproducible.
Randomization	For detection of NETosis markers samples were assigned depending on their attributions as either VITT patients or control samples (all available samples were used). Randomisation was not required as this was not a clinical study. For animal experiments, IgG was isolated from randomly selected patients samples and used in animal experiments. Mice were randomly selected and analysed as biological replicates.
Blinding	Investigators were blinded to group allocation for animal experiments during data collection and analysis. Blinding was not relevant for in vitro studies as this was not a clinical study and knowledge of disease phenotype for sample identification was essential.

Reporting for specific materials, systems and methods

We require information from authors about some types of materials, experimental systems and methods used in many studies. Here, indicate whether each material, system or method listed is relevant to your study. If you are not sure if a list item applies to your research, read the appropriate section before selecting a response.

Materials & experimental systems

n/a	Involved in the study	
☐	☒	Antibodies
☐	☒	Eukaryotic cell lines
☒	☐	Palaeontology and archaeology
☐	☒	Animals and other organisms
☐	☒	Human research participants
☒	☐	Clinical data
☒	☐	Dual use research of concern

Methods

n/a	Involved in the study	
☒	☐	ChIP-seq
☐	☒	Flow cytometry
☒	☐	MRI-based neuroimaging

Antibodies

Antibodies used	Antibody: IV.3; catalog number, IV.3 (ATCC® HB-217™); Supplier, ATCC; clone, HB-217; application, blockage of FcgRIIa in vitro and in vivo.<br>Antibody: anti fibrin; hybridoma supplied by Passam, FH; application, recognition of human fibrin<br>Antibody: AP2; laboratory stock purified from AP2 hybridoma cells; application, anti human CD41<br>Antibody: anti cit H3; catalog number, ab5103; supplier, abcam; clone, polyclonal; application, immunofluorescence, flow cytometry; lot number GR3269686-1, GR3263131-3<br>Antibody: Goat anti-rabbit IgG BV421, 565014; supplier, BD; clone, polyclonal; application, flow cytometry, lot number<br>Antibody: anti CD41 PE 555467 supplier, BD; clone, HIP8; application, flow cytometry; lot number 8218552<br>Antibody: anti CD15 647; catalog number, 562369; supplier, BD; clone, W6D3; application, flow cytometry, lot number 227808, 8249658<br>Antibody: anti MPO; catalog number, 341642; supplier, BD; clone, 5B8; application, flow cytometry; lot number 9191570<br>Antibody: anti-Ly6G; catalog number, 127626; supplier, BioLegend; clone, 1A8; application, immunohistochemistry; B287100<br>Antibody: anti-Ly6G v450; catalog number, 560603; supplier, BD; clone, 1A8; application, flow cytometry; lot number 7165598<br>Antibody: anti CD14; catalog number, 561391; supplier, BD; clone, M5E2; application, flow cytometry; lot number 8245609<br>Antibody: anti CD11b; catalog number, 557397 supplier, BD; clone, M1/70; application, flow cytometry; lot number 9098796<br>Antibody: dylight 649 cd42c; catalog number, X649; supplier, emfret; clone, Xia.C3; lot number 649-D<br>Antibody: CD15 fitc; catalog number, 555401; supplier, BD; clone, HI98; lot number 9017760
Validation	Antibody: IV.3: anti FcgRIIa, effector deficient (in vivo blocking); Validation: Blood. 2015 Nov 5; 126(19): 2230–2238. CD32a

Validation	antibodies induce thrombocytopenia and type II hypersensitivity reactions in FCGR2A mice.<br>Antibody: anti fibrin: anti human and mouse fibrin; validation: J Clin Invest. 2012 Jun 1; 122(6): 2104–2113. Protein disulfide isomerase inhibitors constitute a new class of antithrombotic agents<br>Antibody: AP2: anti human CD41; validation: Blood, vol. 77, pp. 2190 - 2199, Chong BH;Du X;Berndt MC;Horn S;Chesterman CN, 1991, 'Characterization of the binding domains on platelet glycoproteins Ib-IX and IIb/IIIa complexes for the quinine/quinidine dependent antibodies'<br>Antibody: anti cit H3; anti citrullinated histone 3 (human and mouse); validation: flow cyt, human - Gavillet M et al. Flow cytometric assay for direct quantification of neutrophil extracellular traps in blood samples. Am J Hematol 90:1155-8 (2015). WB, Flow Cyt, mouse - Hamaguchi S et al. Origin of Circulating Free DNA in Sepsis: Analysis of the CLP Mouse Model. Mediators Inflamm 2015:614518 (2015).<br>Antibody: anti CD41 PE; anti human CD41; validation: Product website (AUS BD) https://www.bdbiosciences.com/en-au/products/reagents/flow-cytometry-reagents/research-reagents/single-color-antibodies-ruo/pe-mouse-anti-human-cd41a.555467<br>Antibody: anti CD15 647; validation: Product website (AUS BD) https://www.bdbiosciences.com/en-au/products/reagents/flow-cytometry-reagents/research-reagents/single-color-antibodies-ruo/alexa-fluor-647-mouse-anti-human-cd15.562369<br>Antibody: anti MPO; validation: Product website (AUS BD) https://www.bdbiosciences.com/en-au/products/reagents/flow-cytometry-reagents/clinical-discovery-research/single-color-antibodies-ruo-gmp/pe-mouse-anti-human-myeloperoxidase-mpo.341642<br>Antibody: anti-Ly6G; validation: IHC, mouse - Esbona K, et al. 2016. Breast Cancer Res. 18:35.<br>Antibody: anti-Ly6G v450; validation; product website (AUS BD) https://www.bdbiosciences.com/en-au/products/reagents/flow-cytometry-reagents/research-reagents/single-color-antibodies-ruo/v450-rat-anti-mouse-ly-6g.560603<br>Antibody: anti CD14; validation; product website (AUS BD) https://www.bdbiosciences.com/en-au/products/reagents/flow-cytometry-reagents/research-reagents/single-color-antibodies-ruo/v500-mouse-anti-human-cd14.561391<br>Antibody: anti CD11b; validation; product website (AUS BD) https://www.bdbiosciences.com/en-au/products/reagents/flow-cytometry-reagents/research-reagents/single-color-antibodies-ruo/pe-rat-anti-cd11b.557397<br>Antibody: dylight 649 CD42c; validation: "in vivo labelling of platelets, mouse - Falati et al. (2002) Real-time in vivo imaging of platelets, tissue factor and fibrin during arterial thrombus formation in the mouse. Nature Medicine 8, 1175-1181<br>Antibody: CD15 fitc; validation: on product website (AUS BD) https://www.bdbiosciences.com/en-au/products/reagents/flow-cytometry-reagents/research-reagents/single-color-antibodies-ruo/fitc-mouse-anti-human-cd15.555401<br>Antibody: goat anti-rabbit IgG BV421, validation: product website (AUS BD) https://www.bdbiosciences.com/en-au/products/reagents/flow-cytometry-reagents/research-reagents/single-color-antibodies-ruo/bv421-goat-anti-rabbit-igg.565014

Eukaryotic cell lines

Policy information about cell lines

Cell line source(s)	Hybridoma cells expressing IV.3 monoclonal antibody. Catalog number, IV.3 (ATCC® HB-217™); Supplier, ATCC; clone, HB-217
Authentication	Identity of cells based on ATCC documentation and antibody expression. Expression of IV.3 antibody by ATCC® HB-217™ cells against FcgRIIa was confirmed by the authors using flow cytometry with platelets from wild-type mice (which do not express FcgRIIa) and platelets from transgenic mice expressing human FcgRIIa.
Mycoplasma contamination	Hybridoma cells were negative for mycoplasma contamination.
Commonly misidentified lines<br>(See ICLAC register)	No commonly misidentified lines were used in the study.

Animals and other organisms

Policy information about studies involving animals; ARRIVE guidelines recommended for reporting animal research

Laboratory animals	Mus musculus, strain C57/BL6. Two mouse lines: 1) expressing FcgammaRIIa and human PF4 and, 2) expressing FcgammaRIIa and human PF4 and knockout for PAD4. Both males and females, aged 8-12 weeks were used.
Wild animals	No wild animals were used in the study.
Field-collected samples	No field collected samples were used in the study.
Ethics oversight	UNSW Animal Care and Ethics Committee

Note that full information on the approval of the study protocol must also be provided in the manuscript.

Human research participants

Policy information about studies involving human research participants

Population characteristics	The study population comprised subjects diagnosed with vaccine induced thrombosis and thrombocytopenia. Control subjects included healthy participants, vaccinated individuals who did not develop vaccine induced thrombosis and thrombocytopenia and patients with VTE.<br>Population characteristics of patients recruited into the study are described in Supplementary tables 1 and 2 of the manuscript.<br>Covariates in Table 1: site of thrombosis, lab tests and treatment<br>Covariates in Table 2: diagnosis and treatment
Recruitment	Blood samples were collected with informed consent from patients diagnosed with vaccine induced thrombosis and

Recruitment	thrombocytopenia. Controls: blood was taken from healthy subjects (normal), healthy subjects vaccinated with ChAdOx1 nCoV-19 who did not develop VITT (vax), patients with common venous thromboembolism (VTE), heparin-induced thrombocytopenia (HIT), and critically ill patients admitted to intensive care units (ICU), with informed consent. Patients were recruited for the key purpose of sample collection. Although selection bias is possible, it is unlikely to impact results as the population characteristics of each cohort are representative of the Australian population and patients were selected based on clinical diagnosis for VITT, HIT and VTE.
Ethics oversight	The study was approved by the South Eastern Sydney Local Health District Human Research Ethics Committee (17/211 LNR/17/POWH/501)

Note that full information on the approval of the study protocol must also be provided in the manuscript.

Flow Cytometry

Plots

Confirm that:

☒ The axis labels state the marker and fluorochrome used (e.g. CD4-FITC).

☒ The axis scales are clearly visible. Include numbers along axes only for bottom left plot of group (a 'group' is an analysis of identical markers).

☒ All plots are contour plots with outliers or pseudocolor plots.

☒ A numerical value for number of cells or percentage (with statistics) is provided.

Methodology

Sample preparation	Blood was collected from VITT patients and healthy volunteers in sodium citrate or EDTA tubes, as required for the various experiments. Mouse blood was collected into EDTA microvettes. Neutrophils and monocytes were isolated from whole blood using immunomagnetic negative isolation kit and depleted using microbeads. Mononuclear cell layer was isolated using density gradient centrifugation. Samples were diluted with PBS and treated with normal or VITT IgG. Following treatment, cells were stained and fixed prior to flow cytometric analysis.
Instrument	LSRFortessa Cell Analyzer flow cytometer, BD Biosciences
Software	Data collection: FACS DIVA v9, Analysis: FlowJo v10.8.1
Cell population abundance	For human blood FACS analysis, at least a million events were acquired. For platelet count analysis in mice, acquisition was stopped at 60s and counts were calculated relative to time 0 (pretreatment).
Gating strategy	Neutrophil-platelet aggregates and NETs assay: Debris was excluded from the FSC vs SSC profile. Within the neutrophil population (identified as CD15+ cells), the percentage of neutrophil platelet aggregates was classified as CD15+CD41+ events. The percentage of neutrophils undergoing NETosis was classified as CD15+citH3+MPO+ events.<br>Platelet counts: Platelet population gating was determined using log scales for FSC and SSC. Staining with anti CD41 was used to confirm the platelet population.<br>Monocyte and granulocyte population: Debris was excluded from the FSC vs SSC profile. Monocyte (CD11b+Ly6G- cells for mouse; CD14+ cells for human) and neutrophil (CD11b+Ly6G+ cells for mouse, CD15+ cells for human) populations were gated as specified above and backgated to the FSC vs SSC plots. Gating strategies for the subsets of immune cells are as described in Lechner et al., Scientific Reports 5, 16754 (2015).

☒ Tick this box to confirm that a figure exemplifying the gating strategy is provided in the Supplementary Information.
